# Study of Meta-analysis strategies for network inference using information-theoretic approaches

**DOI:** 10.1186/s13040-017-0136-6

**Published:** 2017-05-06

**Authors:** Ngoc C. Pham, Benjamin Haibe-Kains, Pau Bellot, Gianluca Bontempi, Patrick E. Meyer

**Affiliations:** 1 Bioinformatics and Systems Biology (BioSys) Lab, Université de Liège, Liège, Belgium; 2 Princess Margaret Cancer Center, Toronto, ON Canada; 3 Department of Medical Biophysics, University of Toronto, Toronto, ON Canada; 4 Department of Computer Science, University of Toronto, Toronto, ON Canada; 5 Ontario Institute of Cancer Research, Toronto, ON Canada; 6 Image Processing group, Technical University of Catalonia, Barcelona, Spain; 7 Machine Learning Group, Interuniversity Institute of Bioinformatics in Brussels (IB)², Université Libre de Bruxelles, Bruxelles, Belgium

**Keywords:** Meta-analysis, Gene regulatory networks, Systems biology, Gene expression, Mutual information

## Abstract

**Background:**

Reverse engineering of gene regulatory networks (GRNs) from gene expression data is a classical challenge in systems biology. Thanks to high-throughput technologies, a massive amount of gene-expression data has been accumulated in the public repositories. Modelling GRNs from multiple experiments (also called integrative analysis) has; therefore, naturally become a standard procedure in modern computational biology. Indeed, such analysis is usually more robust than the traditional approaches, which suffer from experimental biases and the low number of samples by analysing individual datasets.

To date, there are mainly two strategies for the problem of interest: the first one (“data merging”) merges all datasets together and then infers a GRN whereas the other (“networks ensemble”) infers GRNs from every dataset separately and then aggregates them using some ensemble rules (such as ranksum or weightsum). Unfortunately, a thorough comparison of these two approaches is lacking.

**Results:**

In this work, we are going to present another meta-analysis approach for inferring GRNs from multiple studies. Our proposed meta-analysis approach, adapted to methods based on pairwise measures such as correlation or mutual information, consists of two steps: aggregating matrices of the pairwise measures from every dataset followed by extracting the network from the meta-matrix. Afterwards, we evaluate the performance of the two commonly used approaches mentioned above and our presented approach with a systematic set of experiments based on in silico benchmarks.

**Conclusions:**

We proposed a first systematic evaluation of different strategies for reverse engineering GRNs from multiple datasets. Experiment results strongly suggest that assembling matrices of pairwise dependencies is a better strategy for network inference than the two commonly used ones.

## Background

One of the most long-standing challenges in Systems Biology is the development of methods, which are able to construct the complete set of regulatory interactions of a cell. The regulating circuitry, also called gene regulatory network (GRN), can then be used by bio-medical experts to understand key mechanisms in cells. Thanks to high-throughput technologies, a large amount of transcriptome data is now available through public repositories (e.g. NCBI GEO [1], ArrayExpress [2]), providing opportunities to study the GRNs of many organisms.

In the last decade, a variety of algorithms have been proposed in an attempt to address this *reverse engineering* problem. These algorithms can be classified into several categories [3], such as: regression-based, pairwise similarity (mutual information, correlation,...), Bayesian networks or even ensemble approaches (that combine several different approaches). Among those, mutual information (MI) based algorithms, such as CLR [4], ARACNE [5], MRNET [6, 7] and so on, gather more and more attention owing to their capability to deal with up to several thousands of variables in the presence of a limited number of samples [7]. Generally, MI-based algorithms start by estimating a pairwise mutual information (i.e. a non-linear dependency measure) between all pairs of genes, resulting in a mutual information matrix (MIM). Afterwards, indirect interactions are eliminated from the MIM by the different approaches and subsequently a GRN is inferred.

Since a single dataset has typically a small sample size (usually less than 200 observations) and suffers from potential experimental biases, classical *reverse engineering* algorithms, which relies only on a standalone dataset, show their limits in unravelling reliably underlying interactions. By contrast, integrative analysis of multiple studies is able to increase significantly the statistical power and thus is becoming a standard procedure in modern computational biology [8]. Nevertheless, the question of how to integrate data consistently and efficiently raises new challenges [9].

In the mean time, meta-analysis strategies have been increasingly used for detecting differentially expressed genes from microarray data [10]. In the *meta-analysis* approaches, each single dataset is analysed separately and then the final results are combined [11]. Several strategies have been proposed in order to perform meta-analysis on expression data. For instance, a meta-analysis of public gene expression data and clinical data was conducted by using the concept of “coexpression” modules to reveal various results of previous gene expression studies in breast cancer [12, 13]. In another research [14], Hong et al. developed a Bioconductor package RankProd that allow researchers to do meta-analysis under two experimental microarray conditions to identify differentially expressed genes.

While the problem of detecting differentially expressed genes across several studies has been intensively studied, it is, however, not yet the case when it comes to constructing GRNs.

To deal with the challenge of meta-network inference, there have been plenty of proposed methods, which can be divided into two main categories: “data merging” and “network ensemble”. In the “data merging” approach, datasets are integrated at the expression level into a unique dataset, from which GRNs are inferred [15–17]. However, one of the major problem of this approach is the removal of batch effects. Indeed, the use of different platforms, and different methodologies by different research groups introduce statistical biases (batch effects) that can lead to incorrect conclusions [18]. For example, it is known that normalization techniques, such as RMA [19], consisting in re-scaling gene expression values at the probe intensity level for Affymetrix data [20], is not able to remove batch effects. Consequently, batch removal methods, like COMBAT [21], is typically used before merging data [18].

On the other hand, “ensemble” methods of merging GRNs from different datasets, i.e. by weighting gene-gene interactions according to their average rank in each network [3], have emerged as an alternative to the “data merging” approach. This approach rooted in the “wisdoms of crowds” concept, which was first introduced in the DREAM5 challenge and then further developed by [22] with the Top*k*Net algorithms to produce consensus networks.

In this paper, we also introduce a new meta-analysis strategy to build consensus networks. The new strategy consists in aggregating matrices of pairwise mutual information with each being estimated from a gene expression dataset to produce a meta-matrix, from which a GRN is inferred using classical information-theoretic network inference algorithms. Additionally, the paper presents the first thorough experimental comparison of these three “meta” approaches for the reconstruction of networks, namely “data merging”, “network ensemble” and “coexpression matrices aggregation”. The performances of these three sets of methods are evaluated using synthetic datasets from the standard Bioconductor netbenchmark package.

## Methods

### State-of-the-art

Mutual information is a non-linear measure of dependency between two variables (genes) *X* and *Y*, defined as follow

1 $$ I(X, Y) = \sum\limits_{x\subset X, y\subset Y} p(x, y)log\frac{p(x, y)}{p(x)p(y)}  $$

where *p*(*x,y*) is the joint probability distribution of *X* and *Y*, and *p*(*x*) and *p*(*y*) are the marginal probability distributions of *X* and *Y*, respectively.

This dependency measure has been used for reconstructing networks by several methods such as CLR, ARACNE or MRNET. The first one - CLR method (The Context Likelihood or Relatedness network) [4] creates an edge between each pair of genes *i* and *j* if the combined z-score of the mutual information between them is above a given threshold, where the combined z-score is defined as:

2 $$ c_{ij} = \sqrt{c_{i}^{2} + c_{j}^{2}} \;\text{with}\; c_{i} = \max(0, \frac{M_{ij} - \mu_{M_{i}}}{\sigma_{M_{i}}})  $$

in which, $\mu _{M_{i}}$ and $\sigma _{M_{i}}$ are the mean and standard deviation of the empirical distribution of the mutual information of gene *i*.

The second algorithm named ARACNE (The Algorithm for the Reconstruction of Accurate Cellular Networks) [5] relies on the “Data Processing Inequality” (DPI) which removes the edge with the weakest mutual information, in every triplet of genes.

And finally, the Minimum Redundancy NETworks (MRNET) [6, 7] method reconstructs a network using the feature selection technique known as Minimum Redundancy Maximum Relevance (MRMR) [23]. The minimum redundancy criterion makes the implicit assumption that variables with redundant information to the most relevant variables are indirect links.

**Fig. 1:**
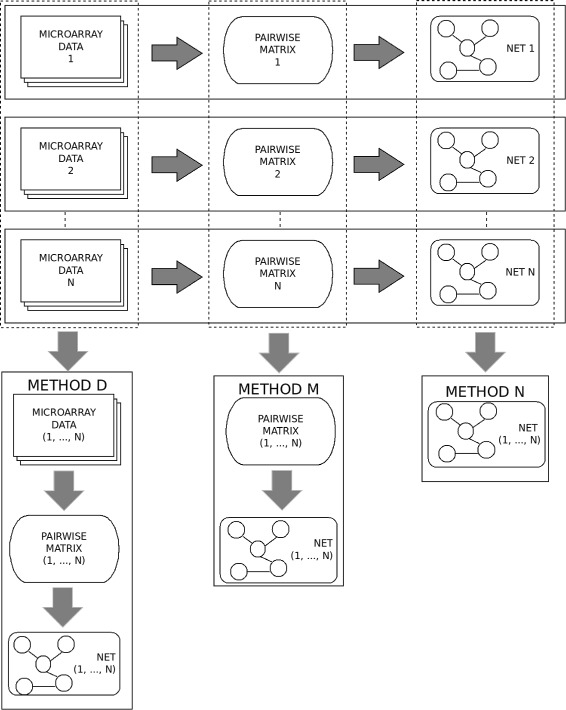
Meta-network strategies: assembling datasets, pairwise matrices or networks

Using these three information-theoretic network inference techniques, which are available from the Bioconductor Minet package, we will evaluate the performance of the three meta-analysis approaches that were demonstrated in Fig. 1 in the next sections.

### Data merging - D methods

A straightforward approach for performing integrative analysis of multiple studies is combining all datasets together and then analysing the merged dataset. These method, named “data merging” and denoted here with the letter (D), were widely used in [15–17] to reconstruct large-scale GRNs because of their simplicity. However, since high dimensional data often suffers from unwanted biases, a variety of techniques can be used to correct for these non-biological variations. We present in the following two classical scaling methods typically used to assemble datasets, and one batch-effect-removal method.

#### Normalization: BMC(D1) and z-score (D2)

Let *X* be a matrix *X* ^*m*×*n*^ denoting the dataset of gene expression values. In this matrix, columns represent samples and rows represent genes, and *x*_*ij*_ represents the expression value of gene *i* in sample *j* of dataset *X*. In [24], a normalization technique named BMC (Eq. 3) was applied for merging breast cancer datasets.

3 $$  \hat{x_{ij}}=x_{ij}-\bar{x_{i}}  $$

Similarly, the z-score normalization [25] is described by Eq. 4 and was also included for evaluation.

4 $$  \hat{x_{ij}}=\frac{x_{ij}-\bar{x_{i}}}{\sigma_{x_{i}}}  $$

#### Batch effects removal: COMBAT(D3)

Gene expression datasets mostly come from different platforms and laboratories, causing the so-called batch effects. Consequently, batch removal methods, like COMBAT (also known as Empirical Bayes) [21], is often used to detect and remove this inevitable variation. COMBAT, which was shown to outperform other commonly used batch removal methods in some specific scenarios [26], uses estimations for the LS (location-scale) parameters (e.x. mean and variance) for each gene independently [27]. The gene, afterwards, is adjusted to meet the estimated model. In this paper, combining datasets using the COMBAT algorithm will be included for comparison and referred as method D3.

### Networks ensemble - N methods

As we presented in the previous subsection, one of the difficulties of the data-merging methods is how to handle the batch effects. Consequently, “networks ensemble” method (denoted with the letter (N) in the paper) has been proposed as an alternative approach. In fact, by combining topologies of networks rather than datasets we are able to avoid dealing with batch effects. This method first constructs every single transcriptional networks independently before combining them to produce a so-called community network [3]. In general, combining networks consists in two distinct steps: transformation and aggregation [28]. Indeed, before assembling networks, a network-normalization step can be performed because it is common to observe networks that exhibit different distribution of edge weights.

Let *e*_*ij*_ be the weight of an edge between gene *i* and gene *j* and *t*_*n*_(*e*_*ij*_) be the normalized value for *e*_*ij*_ in the network *n*. In the next subsections, we discuss three viable combinations of network transformation and aggregation.

#### RankSum method (N1)

The RanSum method, which was introduced in [3], is based on rank averaging: If *e*_*ij*_ denotes an edge connecting genes *i* and *j* and *r*_*n*_(*e*_*ij*_) the rank of the edge in network *n*, the final rank of the edge across *N* networks is computed by:

5 $$  r(e_{ij}) = \sum\limits_{n=1}^{N} {r_{n}(e_{ij})}  $$

#### Internal quality control index (N2)

In [10], six quantitative quality control measures have been proposed for the inclusion/exclusion of gene expression studies used for the meta-analysis. Among these measures, the internal quality control index will be included in this paper, as method N2 for assembling networks. Let the similarity between two studies *m* and *n* be defined as

6 $$  r_{mn}=spcor((t_{n}(e_{ij}); 1 \leq i \leq j \leq G), (t_{m}(e_{ij}); 1 \leq i \leq j \leq G))  $$

In which *r*_*mn*_ is the Spearman’s rank correlation of the pairwise correlation structure between study *m* and *n* and *G* represents the total number of genes in the studies. The dissimilarity (or distance) between study *m* and *n* is defined as *d*_*mn*_=(1−*r*_*mn*_)/2. For a given study *k*, a weight - *w*_*k*_ will be granted as the fraction between the sum of distances between study *k* - $D_{k}^{*}$ to all other studies and the sum of pairwise distances between all studies excluding the study *k* - *Dk* *#* with

7 $$  D_{k}^{*}=\{d_{kn}\}_{1\leq n \leq N; n \neq k} \;\text{and } D_{k}^\#=\{d_{mn}\}_{1\leq m \neq n \leq N; m \neq k; n \neq k}  $$

Afterwards, the weight of the edge between two variables (genes) *X* and *Y* is aggregated by the following equation:

8 $$  \hat{e}_{IQC}(X; Y) = \frac{\sum\limits_{k=1}^{N} {w_{k} t_{k}(e_{XY})}}{\sum\limits_{k=1}^{N} w_{k}}  $$

#### Median method (N3)

In [22] the median value was introduced for aggregating consensus networks. This method assigns the median value among *N* values representing the confidence score of a specific edge in *N* different networks.

9 $$  a_{M}(e_{ij})=median\{t_{1}(e_{ij}), \ldots,t_{N}(e_{ij})\}  $$

### Matrices of coexpression based aggregation approaches - M methods

Our new category of meta-analysis approaches (denoted with the letter (M) in this paper) aggregates mutual information matrices rather than data or networks. The idea behind assembling pairwise matrices is that, although expression data typically shows high variability due to differences in technology, samples, labels, etc., pairwise dependency measures between genes should be much less variant (i.e. dependent variables, such as a regulating variable and its regulated counterpart, should remain dependent in every platform/experiment/dataset even if ranges of values differ greatly). Thus, to infer a network from various expression data, our approach consists in combining mutual information matrices (MIMs) estimated independently from each dataset. Then a GRN network is inferred from the aggregated MIMs. In the following subsections, we will demonstrate three feasible methods to assemble matrices of pairwise measure.

#### Random-effects model (M1)

It should be noted that the problem of combining MIMs across multiple datasets can be framed in the context of a meta-analysis of correlation coefficients [29]. Hunter and Schmidt [30] introduced a single random-effects method based on untransformed correlation coefficients, at which datasets are weighted simply by the sample sizes on which each effect size (the estimated MIM) is based. Our first weighting schema (method M1), described by Eq. 10, utilises this random-effects method, but using MI instead of correlations.

10 $$  \hat{I}_{RE}(X; Y) = \frac{\sum\limits_{k=1}^{N} {n_{k} I(X_{k};Y_{k})}}{\sum\limits_{k=1}^{N} n_{k}}  $$

where *I*(*X*_*k*_;*Y*_*k*_) is the MI between two variable *X*_*k*_ and *Y*_*k*_ in the study *k* and *n*_*k*_ is the number of samples of study *k*.

The idea is simply that effect sizes based on large samples will be more precise than those based on small samples.

#### Internal quality control index (M2)

Here, the internal quality control index measure was used again with some minor modifications. First, the similarity between two studies *m* and *n* was defined as

11 $$  r_{mn}=spcor((I_{mij}; 1 \leq i \leq j \leq G), (I_{nij}; 1 \leq i \leq j \leq G))  $$

Then, the MI between two variables (genes) *X* and *Y* is aggregated by the following equation:

12 $$  \hat{I}_{IQC}(X; Y) = \frac{\sum\limits_{k=1}^{N} {w_{k} I(X_{k};Y_{k})}}{\sum\limits_{k=1}^{N} w_{k}}  $$

#### Median method (M3)

One of the major issue of M1 is that the quality of datasets used in meta-analysis is not explicitly taken into account. Indeed, inclusion of poor quality datasets is likely to weaken statistical power [10]. Thus, an alternative schema for combining MIMs across heterogeneous studies namely method M3 can be proposed. Method M3 is explained by formula 13, in which the aggregated MI of a gene pair *X* and *Y* is the median value of all MI values between them across all studies.

13 $$  \hat{I}_{M}(X, Y) = median(I(X_{1},Y_{1}), I(X_{2},Y_{2}), \ldots I(X_{N},Y_{N}))  $$

## Results

### Simulated datasets

There are two tasks one needs to consider in order to validate networks: 1) defining a “gold standard” - which is a set of true regulations describing the underlying interaction network, 2) selecting quantitative measures to statistically assess the quality of inferred networks. Typically, the first task is addressed by collecting well-known regulations mined from literature with strong supporting evidences. However, those regulations just cover a small part of the underlying network and therefore cannot be an ideal reference network to thoroughly compare methods. Hence the latter approach is often completed by in-silico experiments.

In this paper, in silico benchmarks are selected from every one of the 4 biological networks and artificially generated datasets coming from the Netbenchmark Bioconductor package [31]. The selected datasets are generated by two simulators namely GNW and SynTReN. The GNW simulator generates network structures by extracting parts of known real GRN structures capturing several of their important structural properties while the SynTReN simulator generates the underlying networks by selecting sub-networks from *E. coli* and *Yeast* organisms [31]. The characteristics of the 4 biological networks are presented in more detail in Table 1.

**Table 1:** Networks used in the paper

Network	Name	Topology	Experiments	Genes	Edges
*SynTreN*_300_	S1	E. coli	800	300	468
*SynTreN*_1000_	S2	E. coli	1000	1000	4695
*GNW*_1565_	G1	E. coli	1565	1565	7264
*GNW*_2000_	G2	Yeast	2000	2000	10392

**Fig. 2:**
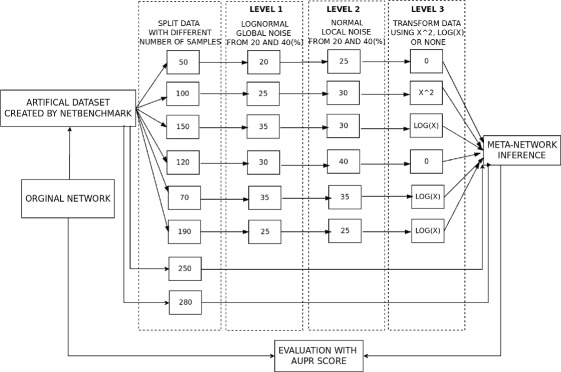
Framework for data collection, network prediction and validation

In the following step, each large dataset will be split into 6 sub-datasets with a number of experiments ranging between 30 to 300 (a number chosen randomly in order to simulate real case scenario where there is a variety of the number of samples). For example, in Fig. 2, an original dataset is split into 6 sub-datasets with the following number of samples: 50, 100, 150, 120, 70 and 190. Additionally, two extremely noisy studies are added, both with a large sample size for each (between 280 and 300). Those datasets allow to test the sensitivity of meta-network methods to datasets that should typically be excluded. Indeed, a few biological studies dating back to the beginning of the microarray technology have very little information and are typically excluded from meta-analysis studies.

### Network prediction and validation for simulated datasets

In order to make the network inference problem more challenging and realistic, noise and transformations of data are added. In particular, we define three levels of data-distortion:

- *i*) Level 1: An independent lognormal noise call “global” noise, with intensity between 20 and 50%, is added to the first 6 datasets. The standard deviation of this noise (*σ*_*Global*_) is the same for the whole dataset and is a percentage (*κ*_*g*_ *%*) of the mean variance of all the genes in the dataset($\bar {\sigma _{g}}$). It is defined as follows: $\sigma _{Global;\kappa _{g}\%} = \bar {\sigma _{g}} \frac {U(0.8\kappa, 1.2\kappa)}{100}$.
- *ii*) Level 2: In addition to the global noise, a normally distributed “local” noise with intensity also ranging between 20 and 50%, is added. This is an additive Gaussian noise with zero mean and a standard deviation (*σ*_*Local*(*g*)_) that is around a percentage (*κ* *%*) of the gene standard deviation (*σ*_*g*_). Therefore, the Signal-to-Noise-Ratio(SNR) of each gene is similar. The local noise standard deviation can be formulated as follows: $\sigma _{Local(g);\kappa \%} = \sigma _{g}\frac {U(0.8\kappa, 1.2\kappa)}{100}$ where *U*(*a,b*) is a uniform distribution between *a* and *b*.
- *iii*) Level 3: In addition to the two previous noises, each sub-dataset can be transformed using a randomly chosen non-linear transformation such as *x* ^2^ or *log*(*x*). This random data transformation is not really meant to be realistic but rather to allow us to better assess the behaviour of each meta-method when faced with extreme distortion. It is worth emphasizing that the two non-informative studies remain unchanged across all experiments. A flowchart of this process is illustrated in Fig. 2.

The schema for network prediction and validation is also illustrated in Fig. 2. Initially, all methods (three D, M and N, totalling nine) are used to construct a consensus GRN from the split datasets. All methods are assessed on 12 challenges (three levels of distortion for four datasets). Finally, the process is repeated for the three information-theoretic inference methods, hence totalling 36 challenges. This is done to make sure that our analysis is not method specific.

Given the ground-truth knowledge of the simulated data, traditional statistical error measures, such as F-score, AUCROC (Area Under the Receiver Operating Characteristic curve) or AUPR (Area Under the Precision-Recall curve) can be used to verify the quality of networks at the global-level [32]. ROC curves, however, can present an overly optimistic view of an algorithm’s performance if there is a large skew in the class distribution [33], which is generally the case in network inference because of its spareness. Consequently, PR curves, which are often used in information retrieval, have been recommended as an alternative to ROC curves [33]. The AUPR for each GRN is, thus, selected to report for all methods in each challenge of the study. Due to the randomization of various experimental parameters (noise intensity, number of samples), 10 repetitions are made. Finally, the average of the ten AUPR values, for each method on each challenge, is presented. Furthermore, in order to see how significantly better is the best method, a p-value using a Wilcoxon test [34] and adjusted, using a Bonferroni correction [35], between each approach and the best one is computed.

### Experimental results

In this section, we present the experimental results of all presented methods for reconstructing GRNs from multiple expression datasets (Table 2). For the D family of methods, it can be observed that normalization using z-score transformation (D2) is better than BMC (D1). This conclusion is true for all three network inference algorithms used in this paper, namely MRNET, ARACNE and CLR. Another striking feature is that batch effect removal methods like COMBAT (D3) is able to increase significantly the robustness of network inference algorithms. The results reinforce the idea that normalization alone can not remove batch effects, and therefore the removal of batch effects is essential when merging datasets. In the case of method N, N2 and N3 outperform N1 when MRNET or CLR used. However, in the case of using ARACNE, N1 is as good as N2 while poor results are observed for N3.

**Table 2:** Area under PR-Curves (the higher the better) for 9 methods on 4 datasets with 3 levels of increasing data-distortion

**MRNET**		D1	D2	D3	N1	N2	N3	M1	M2	M3
***S1***	Level 1	0.082	0.116	0.107	0.052	0.138	0.124	**0.141**	0.137	0.121
	Level 2	0.078	0.110	0.101	0.051	0.119	0.116	**0.120**	0.117	0.102
	Level 3	0.088	0.099	0.096	0.050	0.116	0.114	**0.120**	0.112	0.105
***S2***	Level 1	0.013	0.016	0.016	0.023	0.034	0.026	**0.046**	0.043	0.026
	Level 2	0.013	0.016	0.016	0.024	0.023	0.021	**0.026**	0.025	0.019
	Level 3	0.014	0.016	0.016	0.024	0.024	0.021	**0.027**	0.025	0.020
***G1***	Level 1	0.051	0.099	0.122	0.051	0.125	0.129	**0.156**	0.142	0.131
	Level 2	0.037	0.087	0.108	0.049	0.108	0.115	**0.138**	0.122	0.116
	Level 3	0.039	0.077	0.101	0.048	0.104	0.113	**0.133**	0.115	0.111
***G2***	Level 1	0.028	0.050	0.073	0.029	0.106	0.097	**0.131**	0.126	0.097
	Level 2	0.023	0.046	0.066	0.028	0.089	0.084	**0.116**	0.111	0.085
	Level 3	0.029	0.044	0.066	0.028	0.088	0.085	0.113	0.111	0.087
**Mean**		0.041	0.065	0.074	0.038	0.090	0.087	**0.106**	0.099	0.085
***p*** **-value**		.00195	.00195	.00195	.00195	.00195	.00195		.00195	.00195
**ARACNE**										
***S1***	Level 1	0.032	0.043	0.042	0.045	**0.101**	0.030	0.063	0.055	0.051
	Level 2	0.034	0.042	0.040	0.036	**0.080**	0.022	0.045	0.046	0.039
	Level 3	0.038	0.039	0.038	0.038	**0.083**	0.023	0.049	0.049	0.047
***S2***	Level 1	0.005	0.005	0.006	0.017	0.020	0.006	**0.025**	0.022	0.013
	Level 2	0.005	0.005	0.005	**0.015**	**0.015**	0.005	0.014	0.013	0.009
	Level 3	0.005	0.005	0.005	**0.015**	**0.015**	0.005	0.013	0.012	0.008
***G1***	Level 1	0.030	0.061	0.083	**0.126**	0.119	0.075	0.131	0.116	0.102
	Level 2	0.022	0.054	0.071	0.102	0.092	0.056	**0.105**	0.090	0.087
	Level 3	0.025	0.047	0.068	0.105	0.096	0.058	**0.109**	0.096	0.086
***G2***	Level 1	0.013	0.028	0.048	0.096	0.095	0.052	**0.124**	0.116	0.090
	Level 2	0.010	0.023	0.036	0.068	0.065	0.032	**0.081**	0.075	0.061
	Level 3	0.011	0.018	0.035	0.070	0.070	0.034	**0.087**	0.084	0.058
**Mean**		0.019	0.031	0.040	0.061	**0.071**	0.033	0.070	0.064	0.054
***p*** **-value**		.00195	.00195	.00195	.00977	1.0	.00195		.00586	.00195
**CLR**										
***S1***	Level 1	0.116	0.138	0.136	0.051	0.134	0.130	**0.137**	0.135	0.136
	Level 2	0.122	**0.140**	0.138	0.051	0.135	0.132	0.138	0.137	0.136
	Level 3	0.123	0.131	0.133	0.049	0.135	0.131	**0.138**	0.137	0.136
***S2***	Level 1	0.034	0.042	**0.043**	0.024	0.042	0.040	**0.043**	0.042	0.042
	Level 2	0.032	0.042	**0.043**	0.025	0.041	0.039	**0.043**	0.042	0.042
	Level 3	0.035	0.041	0.042	0.024	0.042	0.039	**0.043**	**0.043**	0.042
***G1***	Level 1	0.062	0.136	0.147	0.047	0.129	0.112	**0.147**	0.138	0.145
	Level 2	0.067	0.135	0.145	0.046	0.126	0.106	**0.142**	0.126	0.138
	Level 3	0.065	0.111	0.132	0.046	0.119	0.104	**0.135**	0.124	0.134
***G2***	Level 1	0.042	0.081	0.095	0.026	0.090	0.078	**0.105**	0.100	0.104
	Level 2	0.041	0.078	0.091	0.026	0.083	0.072	**0.097**	0.095	0.095
	Level 3	0.042	0.066	0.084	0.026	0.081	0.069	**0.094**	0.091	0.093
**Mean**		0.065	0.095	0.102	0.037	0.096	0.088	**0.105**	0.101	0.103
***p*** **-value**		.00195	.00195	.06446	.00195	.00195	.00195		.00195	.00195

**Fig. 3:**
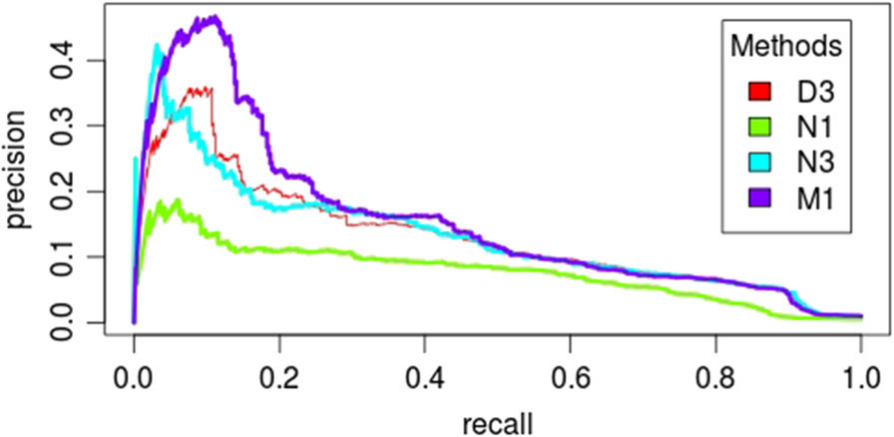
PR-Curves of method D3, N1, N3 and M1 on dataset S1 at level 1 of data distortion

**Fig. 4:**
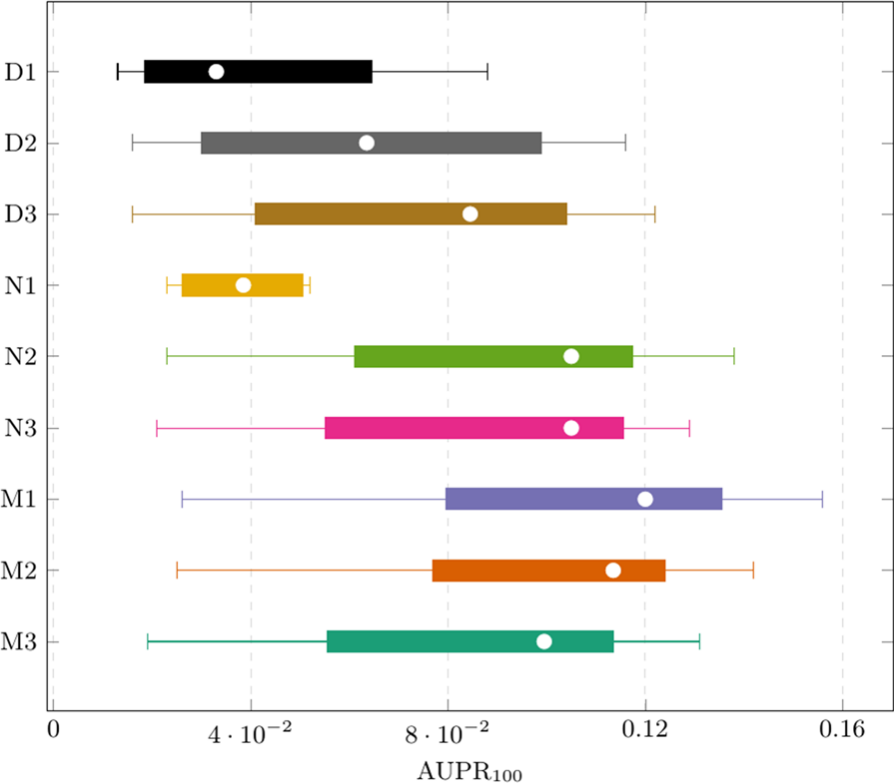
Boxplots for presented methods using MRNET

Interestingly, we can clearly observe that N2 outperforms all three D methods suggesting that assembling networks is better than merging datasets. This could be explained by the fact that gene expression values are very dissimilar in various experiments due to our simulated batch effects (i.e. datasets with different global and local noise). However, the particular combination CLR - D3 offers an exception to this observation. It also should be noted that assembling mutual information matrices (M methods) surpasses the two other well-known strategies (D and N) for all datasets under every different levels of distortion, in particular for MRNET (see Figs. 3 and 4) and CLR. Experimental results also show that MRNET benefits the most from meta-analysis and CLR appears to be the most robust. This suggests that while CLR might be a better strategy for analysing individual datasets, MRNET might be a better choice when multiple datasets are available. Although ARACNE appears to be much worse than the two other techniques, that is mainly due to a bad recall (though not visible with AUPR numbers, its precision remains quite competitive). Finally, in the M family of methods, it appears that combining MIM using random effect model (M1) is better than the two other strategies, the internal quality control index (M2) and the median method (M3).

## Conclusion

In the present paper, we proposed a framework for evaluating the different strategies for inferring GRNs from multiple expression datasets. To the best of our knowledge, this is the first systematic evaluation of the two state-of-the-art strategies for the problem of interest, namely “data merging” and “networks ensemble”. Furthermore, we presented a new, but promising approach for methods based on coexpression matrices. Indeed, our set of experiments strongly suggest that assembling matrices of pairwise dependencies is a better strategy for network inference than the two commonly used ones. However, there exists many different methods of data and network assembly, as well as experimental conditions that have still to be tested in order to gain a complete understanding of the problem of meta-network inference. Moreover, as mentioned earlier, a large amount of under-exploited transcriptome data of model organisms is now available through public repositories. Thus, additionally to testing new ensemble methods, future works include the use of the best strategy to reconstruct large-scale GRNs of these model organisms.

## Abbreviations

AUPR: Area under precision recall
GEO: Gene expression Omnibus
GRNs: Gene regulatory networks
MIM: Mutual information matrix
